# Origin of Electrochemical Activation Leading to Enhanced Cycling Stability of Li‐ and Mn‐Rich Cathodes

**DOI:** 10.1002/anie.8818196

**Published:** 2026-03-23

**Authors:** Peng Zuo, Daniel P. Abraham, Chongmin Wang

**Affiliations:** ^1^ Environmental Molecular Sciences Laboratory Pacific Northwest National Laboratory Richland Washington USA; ^2^ Chemical Sciences and Engineering Division Argonne National Laboratory Lemont Illinois USA

**Keywords:** electrochemical activation, Li_2_MnO_3_ type *C2/m* domains, Li‐rich and Mn‐rich cathode, spinel‐like phase

## Abstract

Electrochemical activation is a critical step for optimal functioning of Li‐ and Mn‐rich (LMR) cathodes, yet the underlying mechanism for such activation remains elusive. Here, by using scanning/transmission electron microscopy (S/TEM) combined with the associated energy‐dispersive x‐ray spectroscopy (EDS) and electron energy‐loss spectroscopy (EELS), we decipher the origin of the activation enhanced electrochemical properties. We reveal that activation induces the formation of a spinel‐like phase within the *C2/m* domains of the LMR cathode, where the transition‐metal ions partially occupy both the tetrahedral (8a) and octahedral (16c) sites of the *Fd* $\bar{3}$ *m* spinel lattice, distinguishing the spinel‐like phase from the conventional high‐voltage spinel. Systematic varying the cycling voltage reveals a critical activation voltage above which this spinel‐like phase forms, while lower voltages preserve the layered bulk structure. As the spinel‐like phase is a stable structure for electrochemical cycling, the present findings provide direct mechanistic insight into the voltage‐dependent activation process and explain how the *C2/m* to spinel‐like transformation upon activation contributes to the electrochemical performance of LMR cathodes, providing guidance for the rational design of Li‐rich cathodes with enhanced cycling durability.

## Graphical abstract

Electrochemical activation is critical for enhancing the performance of Li‐ and Mn‐rich (LMR) cathodes, however, the underlying mechanism has not been fully understood. A spinel‐like phase is found within the *C2/m* domains of LMR after activation, where above a critical activation voltage is needed.

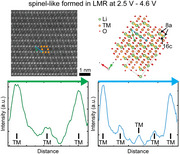

## 1 Introduction

Li‐rich and Mn‐rich (LMR) oxides are a promising class of cathode materials for Li‐ion batteries because they combine high specific capacities (> 200 mAh/g) with the use of earth‐abundant transition metals, particularly manganese [1, 2, 3, 4, 5]. The electrochemical performance of LMR materials strongly depends on their phase composition and microstructure [1, 6, 7, 8, 9]. It is established that LMR cathodes consist of a composite layered structure comprising Li_2_MnO_3_‐type (*C2/m* symmetry) and α‐NaFeO_2_‐type (*R* $\bar{3}$ *m* symmetry) domains [10, 11, 12, 13, 14, 15]. Both structures share a face‐centered cubic oxygen sublattice, with cations occupying octahedral sites arranged in alternating Li‐ and TM‐ rich slabs. However, in the Li_2_MnO_3_‐type structure, excess Li ions also occupy sites within the TM layers, providing a key structural signature distinguishing it from the *R* $\bar{3}$ *m* phase [15]. Furthermore, introducing a spinel component into layered cathodes, forming a spinel‐layered composite, has been shown to improve electrochemical performance by enhancing cycling stability and rate capability [16, 17, 18, 19, 20, 21, 22, 23].

A high‐voltage activation process (typically above 4.5 V) is routinely applied to LMR cathodes during initial cycles to achieve optimal electrochemical performance [24, 25]. This activation step is believed to make the *C2/m* (Li_2_MnO_3_‐type) domain, which is nominally electrochemically inactive, become active [26, 27]. Prior studies have reported the formation of a LiMn_3_O_4_‐like defect spinel in Li_2_MnO_3_ after electrochemical activation between 2.0 and 4.8 V at 50°C [6]. However, the atomic‐scale mechanism of phase formation and its voltage dependence during activation in LMR composite cathodes remain unclear. This lack of understanding hampers efforts to optimize activation conditions and increase cycling stability of the LMR cathodes.

In this study, we use scanning/transmission electron microscopy (S/TEM) combined with the associated energy‐dispersive x‐ray spectroscopy (EDS) and electron energy‐loss spectroscopy (EELS) to investigate the phase evolution of an LMR cathode down to the atomic scale with a nominal composition 0.3Li_2_MnO_3_·0.7LiMn_0.5_Ni_0.5_O_2_ (Li_1.13_Mn_0.57_Ni_0.3_O_2_) during electrochemical activation. We find that a spinel‐like structure form within the *C2/m* domain after activation at 4.6 V. To distinguish between the spinel‐like phase and the spinel phase, in the spinel‐like phase TM ions partially occupy both the tetrahedral (8a) and octahedral (16c) sites of the *Fd* $\bar{3}$ *m* spinel structure, whereas the former sites are occupied by Li ions and the latter remains vacant in the high‐voltage spinel. Systematic comparison across different activation voltages reveals that a critical activation voltage exists for the formation of this spinel‐like phase. Overall, our findings provide direct atomic‐scale evidence for a *C2/m* to spinel‐like phase transformation during activation and highlight the essential role of activation voltage in determining LMR cathode structure and cycling stability. This mechanistic insight clarifies the function of activation in enabling sustained electrochemical performance and offers design guidance for improving next‐generation Li‐rich and Mn‐rich cathodes.

## 2 Results and Discussions

### 2.1 Spinel‐Like Phase Formation Upon Electrochemical Activation

**FIGURE 1:**
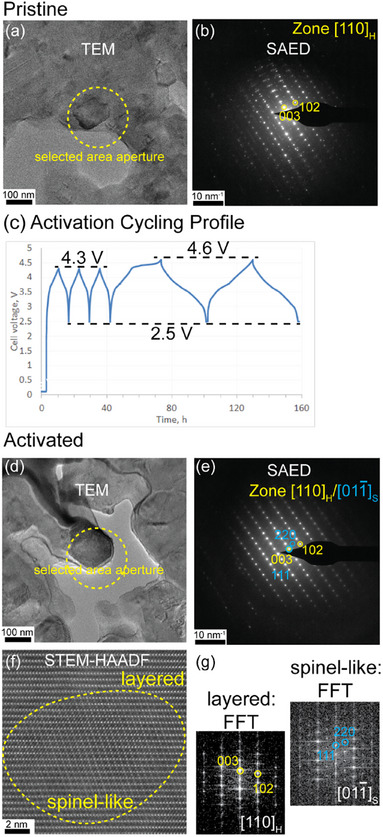
Comparison of phase formation in the pristine and electrochemically activated LMR cathodes at the high cut‐off voltage 4.6 V. (a) TEM image of a pristine LMR particle and (b) corresponding SAED pattern (acquired area highlighted by the yellow circle in (a), indicating only layered phases. (c) Electrochemical activation profile. (d) TEM image of an activated LMR particle and (e) the corresponding SAED pattern, showing the emergence of a spinel‐like phase alongside the layered structure after activation at the high cut‐off voltage 4.6 V. (f) STEM‐HAADF image revealing the coexistence of spinel‐like and layered phases. (g) FFT patterns obtained from the two phases for comparison.

In the pristine state (Figure 1a,b), the LMR37 particles exhibit a layered structure composed of *R* $\bar{3}$ *m* and *C2/m* domains, as detailed in a previous publication [15]. The TEM selected‐area electron diffraction (SAED) pattern can be indexed to the layered *R* $\bar{3}$ *m* structure viewed along the [110]_H_ zone axis, where the subscript “H” denotes the hexagonal structure of the *R* $\bar{3}$ *m* phase. Along this orientation, the *R* $\bar{3}$ *m* and *C2/m* structures cannot be distinguished. The additional diffraction spots in Figure 1b arise from neighboring particles included within the selected‐area aperture, as seen in the corresponding TEM image (Figure 1a).

Upon electrochemical activation (Figure 1c), a new set of diffraction spots appear, which can be indexed to a cubic spinel phase, as shown in Figure 1e. These reflections correspond to the cubic spinel structure viewed along the [01$\bar{1}$]_S_ (“S” denotes the cubic spinel structure) zone axis, where the (111)_S_ reflection of the spinel phase overlaps with the (003)_H_ reflection of the layered *R* $\bar{3}$ *m* structure. Given that both the cubic spinel and the layered structures adopt a face centered cubic (FCC) packing of the oxygen sublattice, the crystal orientations between the cubic spinel and layer structure can be written as [0001]_H_ || [111]_S_ and [$\bar{1}$100]_H_ || [01$\bar{1}$]_S_, respectively. This orientation relationship indicates that the oxygen sublattices of the two phases are aligned and continuous across the interface. The difference between the phases therefore arises from the distinct ways in which cations occupy sites within the shared oxygen framework.

The STEM‐HAADF image in Figure 1f clearly reveals the coexistence of multiple phases within a single particle, each exhibiting distinct atomic arrangements, as further illustrated by the corresponding FFT patterns in Figure 1g. Detailed atomic‐scale analysis of the STEM‐HAADF images shows that the newly formed phase is spinel‐like, rather than a conventionally known cubic spinel phase such as the high‐voltage spinel LiNi_x_Mn_2‐x_O_4_ [28]. To clarify this distinction, Figure S1 compares the ideal spinel and the spinel‐like structures discussed here. In the conventional spinel structure (*Fd* $\bar{3}$ *m*), Li ions occupy tetrahedral 8a sites and TM occupy octahedral 16d sites [29]. In the spinel‐like structure, however, the normally vacant 16c octahedral sites in the spinel structure are partially occupied, and a mixing of Li and TM could occur at both the 8a and 16d positions.

**FIGURE 2:**
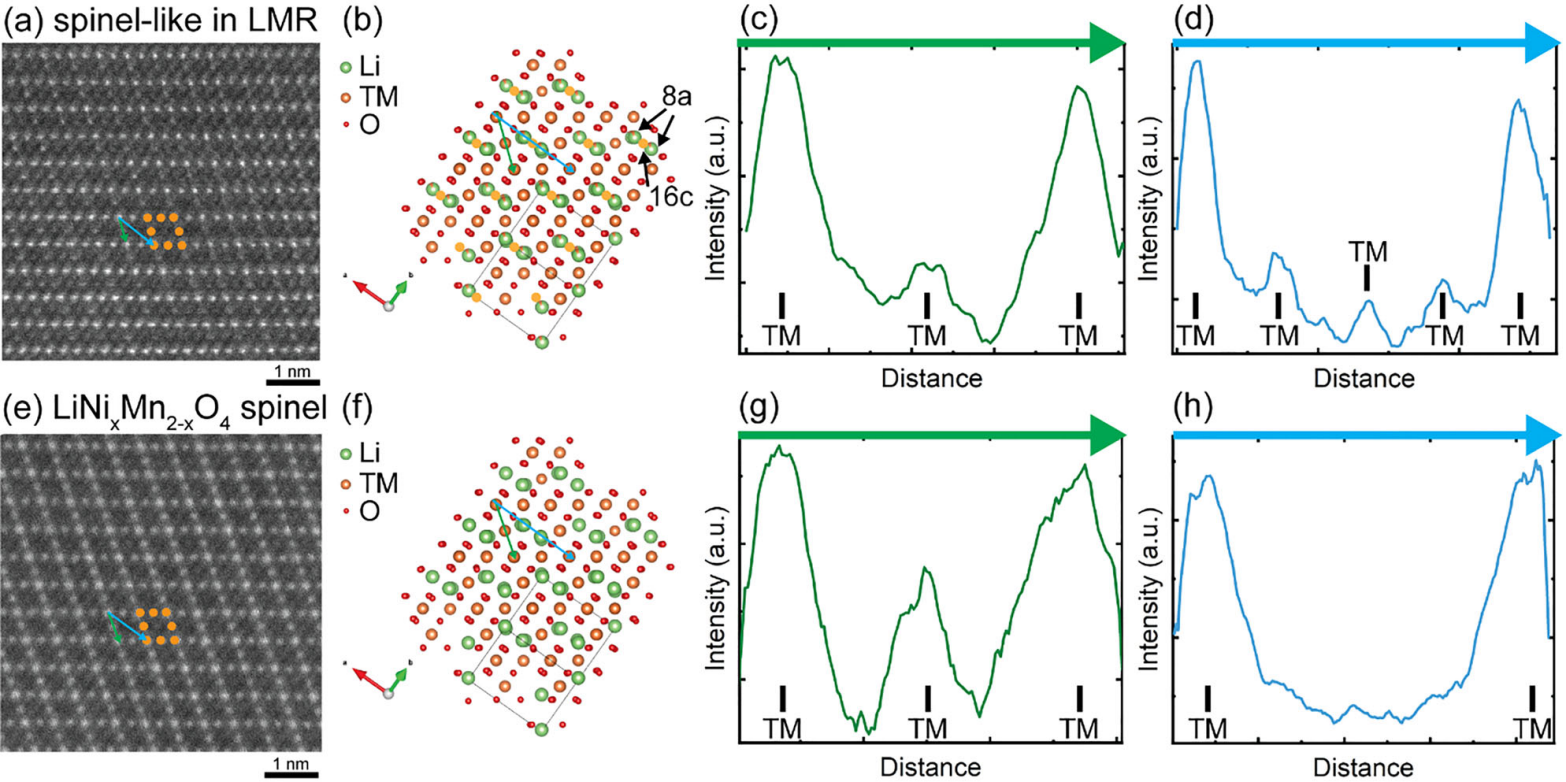
Comparison of atomic structures of the electrochemically induced spinel‐like phase and the high‐voltage spinel phase. (a) STEM‐HAADF image of the spinel‐like phase formed after activation at the high cut‐off voltage 4.6 V with (b) corresponding structural model; (c and d) intensity profiles along the green and blue arrows in (a). (e) STEM‐HAADF image of the pristine high‐voltage spinel with (f) its structural model; (g and h) intensity profiles along the corresponding green and blue arrows in (e).

Figure 2 compares the atomic arrangements of the electrochemically induced spinel‐like phase and the high‐voltage spinel LiNi_x_Mn_2‐x_O_4_ using intensity profiles of the STEM‐HAADF images. Both phases are aligned along equivalent crystallographic orientations, where the image intensities are measured along the equivalent orientations as indicated by the green and blue arrows in Figure 2. The intensity profiles reveal that the central position along the green arrow in the spinel‐like structure exhibits lower intensity than the corresponding position in the high‐voltage spinel structure. More importantly, measurable intensities appear at the 8a and 16c sites (*Fd* $\bar{3}$ *m*) in the spinel‐like phase, whereas no intensities arising from these positions in high‐voltage spinel LiNi_x_Mn_2‐x_O_4_. In the latter, the 8a sites are occupied by Li (a light element), and the 16c sites remain vacant (Figure 2b). Because the STEM‐HAADF contrast scales roughly with the square of the atomic number [30], light elements such as Li and vacancies contribute minimal intensity. The presence of measurable intensities at these positions in Figure 2a thus proves that the electrochemically induced phase is spinel‐like rather than a stoichiometric lithiated cubic spinel. A structural model of the spinel‐like phase is shown in Figure 2a for comparison with the high‐voltage spinel structure in Figure 2b. It should be noted that, because STEM imaging represents a projection through a finite sample thickness, the apparent cation occupancy at the 16c sites does not necessarily imply full occupancy in every unit cell.

Interestingly, formation of spinel‐like phases has also been reported during electrochemical cycling of lithium‐rich disordered rock‐salt (DRX) cathodes [31, 32, 33]. The spinel‐like transformation observed here, and in those studies, suggests that electrochemical cycling drives comparatively low‐symmetry layered (*C2/m*: 11; *R* $\bar{3}$ *m*: 166) and rock‐salt (*Fm* $\bar{3}$ *m*: 225) structures toward a higher‐symmetry spinel arrangement (*Fd* $\bar{3}$ *m*: 227). This evolution likely reflects the greater thermodynamic stability of the spinel‐like arrangement during cycling. Indeed, considerable research is currently directed toward direct synthesis or controlled formation of spinel‐like phases to enhance long‐term structural and electrochemical stability of the battery [32].

### 2.2 Critical Activation Voltage for the Spinel‐Like Phase Formation

**FIGURE 3:**
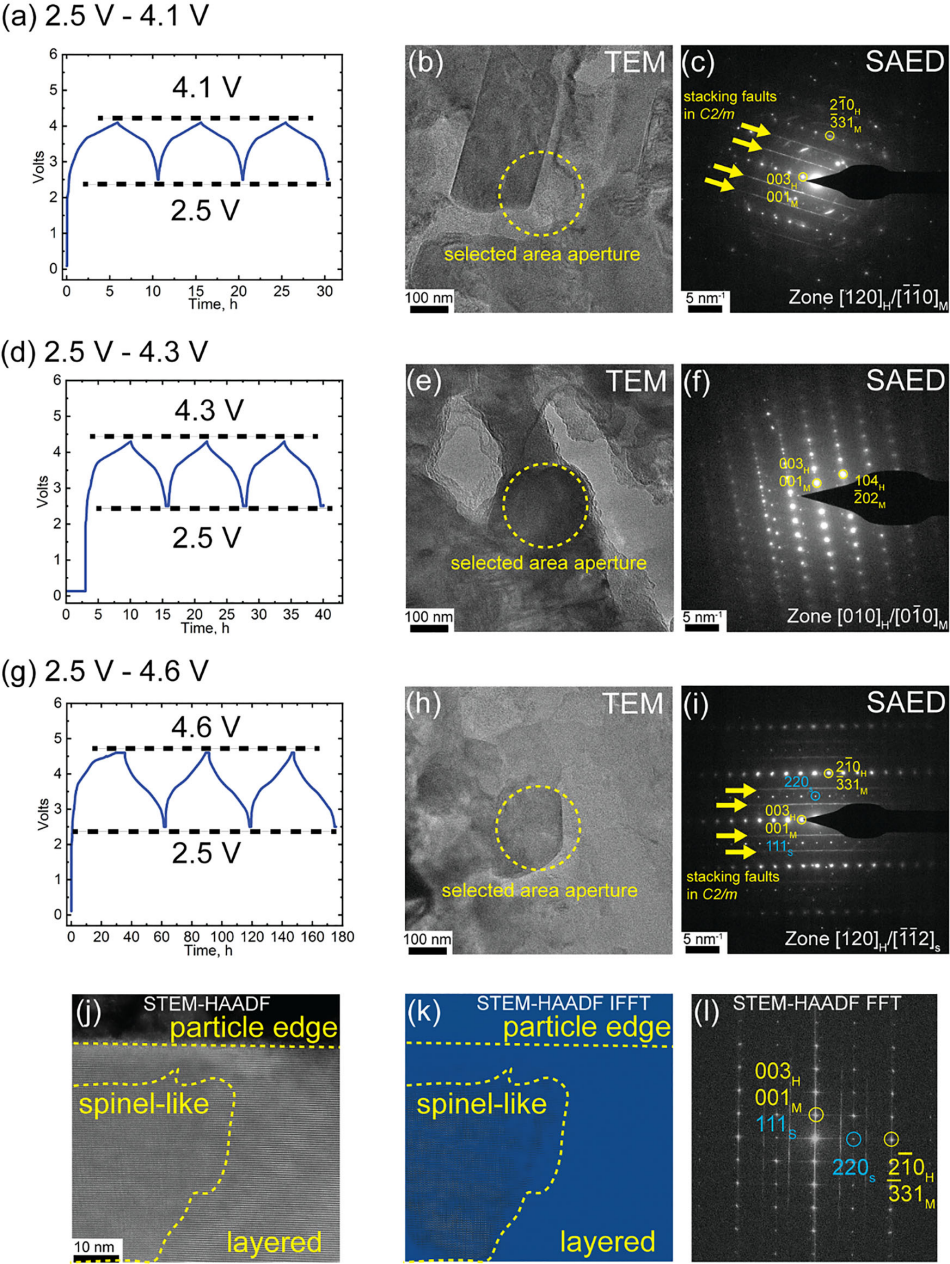
Effect of activation voltage on the formation of spinel‐like phase in layered LMR cathodes during the first three cycles. (a–c) Cycling profile, TEM image, and SAED pattern for 2.5–4.1 V cycling; (d–f) corresponding data for 2.5–4.3 V; (g–i) corresponding data for 2.5–4.6 V, showing emergence of spinel‐like features in the SAED patterns; (j) STEM‐HAADF image showing coexistence of layered and spinel‐like phases; (k) IFFT image revealing spatial phase distribution; (l) FFT pattern indexed to both layered and spinel‐like phases. It should be noted that comparing (a), (d), (g), the cycling time in (g) is much longer. This is because the cell capacities (mAh) are much greater. Because the cycling current (mAh) is the same for all cells, the cycling time (h) is greater for the (4.6 V) cell with the higher capacities.

The effect of activation voltage on the spinel‐like phase formation was examined by varying the upper cut‐off voltages during initial cycling, specifically 4.1, 4.3, and 4.6 V, while maintaining the same three cycles in each case. As seen in the SAED patterns of Figure 3, the spinel‐like phase is observed only in samples cycled to 4.6 V, whereas samples cycled to 4.1 or 4.3 V remain fully layered. This finding indicates that the layered to spinel‐like transformation is strongly dependent on the activation voltage, consistent with literature reports showing that activation above approximately 4.5 V is required to trigger the structural transition in Li‐ and Mn‐rich layered oxides [24, 25]. The STEM‐HAADF image in Figure 3j shows the coexistence of layered and spinel‐like domains within a single particle, and the corresponding inverse fast Fourier transform (IFFT) image clearly visualizes the spatial distribution of these phases (Figure 3k).

**FIGURE 4:**
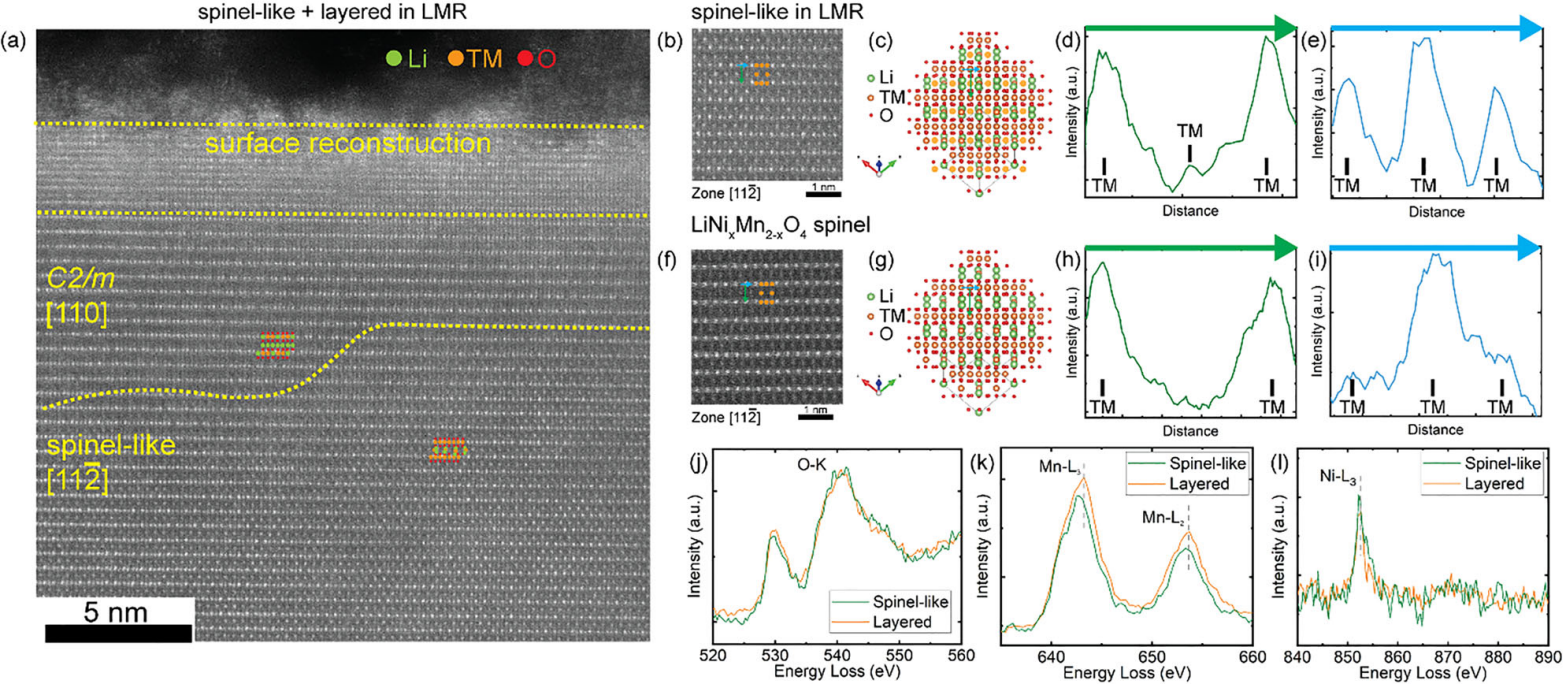
Atomic and electronic structure of the spinel‐like phase formed after three cycles of 2.5–4.6 V. (a) STEM‐HAADF image showing adjacent spinel‐like and layered *C2/m* domains; (b–d) STEM‐HAADF image, structural model, and corresponding intensity profile of the spinel‐like phase along the[11$\bar{2}$] zone axis; (f–h) analogous data for the high‐voltage spinel LiNi_x_Mn_2‐x_O_4_; (j–l) comparison of STEM‐EELS spectra between the spinel‐like and layered phases: (j) O–K edge, (k) Mn‐L_2,3_ edges, and (l) Ni‐L_2,3_ edges.

Detailed structural analysis further indicates that the spinel‐like phase originates primarily from the *C2/m* domains of the LMR cathode. As shown in Figure 4a, the layered region adjacent to the spinel‐like domain displays *C2/m*‐type ordering, suggesting that the transformation proceeds preferentially from the *C2/m* phase rather than from the *R* $\bar{3}$ *m* domains. This observation agrees with previous reports of spinel formation in Li_2_MnO_3_‐type materials after electrochemical activation [6].

High‐resolution STEM‐HAADF imaging and intensity analysis were performed along the [11$\bar{2}$] zone axis, another high‐symmetry orientation of the spinel structure (Figure 4b–d). Despite the apparent similarity in atomic arrangements between the spinel‐like and high‐voltage spine LiNi_x_Mn_2‐x_O_4_ phases in the STEM‐HAADF images, the measured intensity profiles reveal clear differences in site occupancies. Specifically, the octahedral 16c sites of the *Fd3̅m* spinel, which should be vacant in the ideal structure, are partially occupied by TM ions in the spinel‐like phase. This observation is consistent with the analysis along the [01$\bar{1}$] direction shown in Figure 2, confirming that the spinel‐like structure incorporates TM ions into normally vacant positions, producing local cation disorder and partial deviation from the ideal spinel stoichiometry.

Complementary STEM‐EELS analyses were performed to examine the local chemical environment of the spinel‐like and layered phases (Figure 4j–l). The O–K edge spectra (Figure 4j) exhibit a decreased pre‐edge intensity in the spinel‐like region, indicating a comparative oxygen deficiency. Correspondingly, the Mn‐L_2,3_ edges (Figure 4k) shift toward lower energy loss in the spinel‐like phase, suggesting partial reduction of Mn, whereas the Ni‐L_2,3_ edges (Figure 4l) show no significant chemical shift.

Taken together, the consistent observation of the spinel‐like phase after activation at 4.6 V, but not at lower voltages, demonstrates that the structural transformation is primarily governed by the activation voltage rather than the number of initial cycles. TM migration especially the highly mobile Mn to the spinel tetrahedral (8a) and octahedral (16c) sites is a key factor in forming the spinel‐like phase, which is facilitated by the activation of oxygen redox process, therefore accounting for the observation of critical voltage for such a phase transition to happen [32].

### 2.3 Activation Voltage Affects Oxygen Loss and Transition Metal Reduction at Particle Surfaces

**FIGURE 5:**
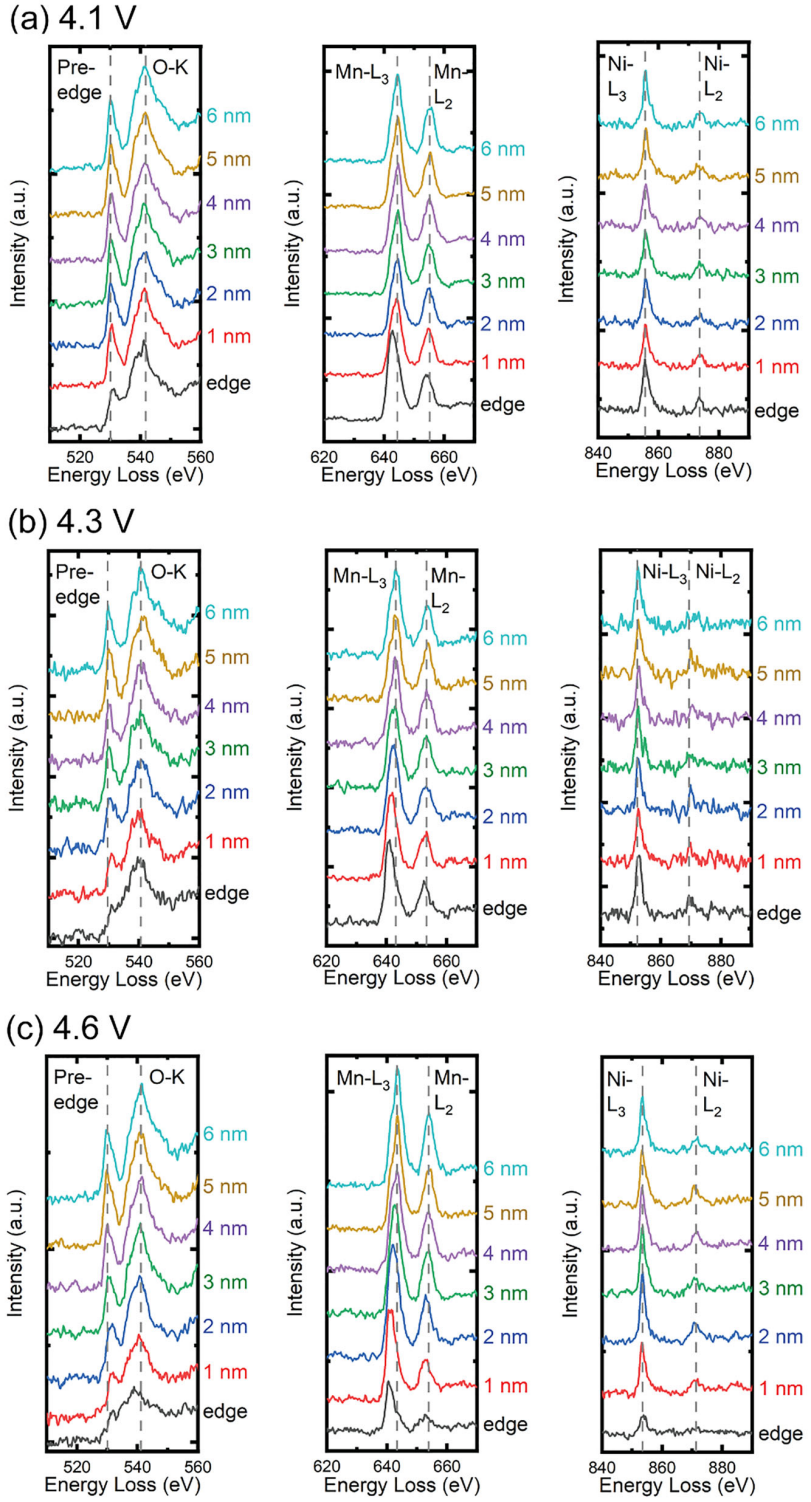
STEM‐EELS analysis of the O‐K edge, Mn‐L_2,3_ edges and Ni‐L_2,3_ edges collected from the particle edge regions of LMR37 electrodes after three initial cycles between (a) 2.5–4.1 V, (b) 2.5–4.3 V and (c) 2.5–4.6 V.

Surface oxygen loss accompanied by TM reduction has been widely reported for layered oxide cathodes [34, 35, 36]. Here, we demonstrate that the extent of surface oxygen loss in cycled LMR cathodes is strongly influenced by the electrochemical activation voltage. In STEM‐EELS analysis, oxygen content can be inferred from two features: intensity of the O–K edge pre‐peak and oxidation state of TM ions. Oxygen loss leads to a decrease to the O–K edge pre‐peak intensity, accompanied by TM reduction. Figure 5 compares STEM‐EELS spectra acquired as a function of distance from the particle surface to the interior after cycling between 2.5–4.1, 2.5–4.3, and 2.5–4.6 V, respectively. The O–K edge pre‐peak clearly shows oxygen depletion within the near‐surface region (∼1 nm depth). Furthermore, the degree and depth of oxygen loss depend on the cycling voltage: higher activation voltages result in more pronounced oxygen loss extending deeper into the particle, as indicated by the progressive decrease in pre‐peak intensity near the surface (Figure S2). The intensity ratio solely is not conclusive as the error bars are hard to estimate. Combining with the chemical shift of the O–K edge toward higher energy loss as the probe moves from the surface to the interior, the more serious oxygen loss with increasing activation voltage is conclusive and consistent with prior reports of surface oxygen depletion [37].

The voltage‐dependent oxygen loss is accompanied by a reduction in the Mn oxidation state. For the particle cycled to 4.1 V, the Mn‐L_3_ edge exhibits a chemical shift of ∼1.8 eV toward lower energy at the surface, decreasing to a shift of ∼0.4 eV at a distance of ∼1 nm from the particle surface. For 4.3 V cycling, the Mn‐L_3_ shift increases to ∼2.2 eV at the surface and a shift of ∼1.4 eV at 1 nm depth. At 4.6 V, the Mn‐L_3_ shift reaches ∼3.0 eV at the surface and a shift of ∼2.4 eV at 1 nm depth, indicating progressively greater Mn reduction near the surface with increasing voltage. In contrast, the Ni‐L_2,3_ edges show no chemical shift (Figure 5), suggesting that Ni oxidation states are less affected by cycling voltage under these conditions.

### 2.4 Activation Voltage Dependence of Cathode–Electrolyte Interphase Layer Formation

**FIGURE 6:**
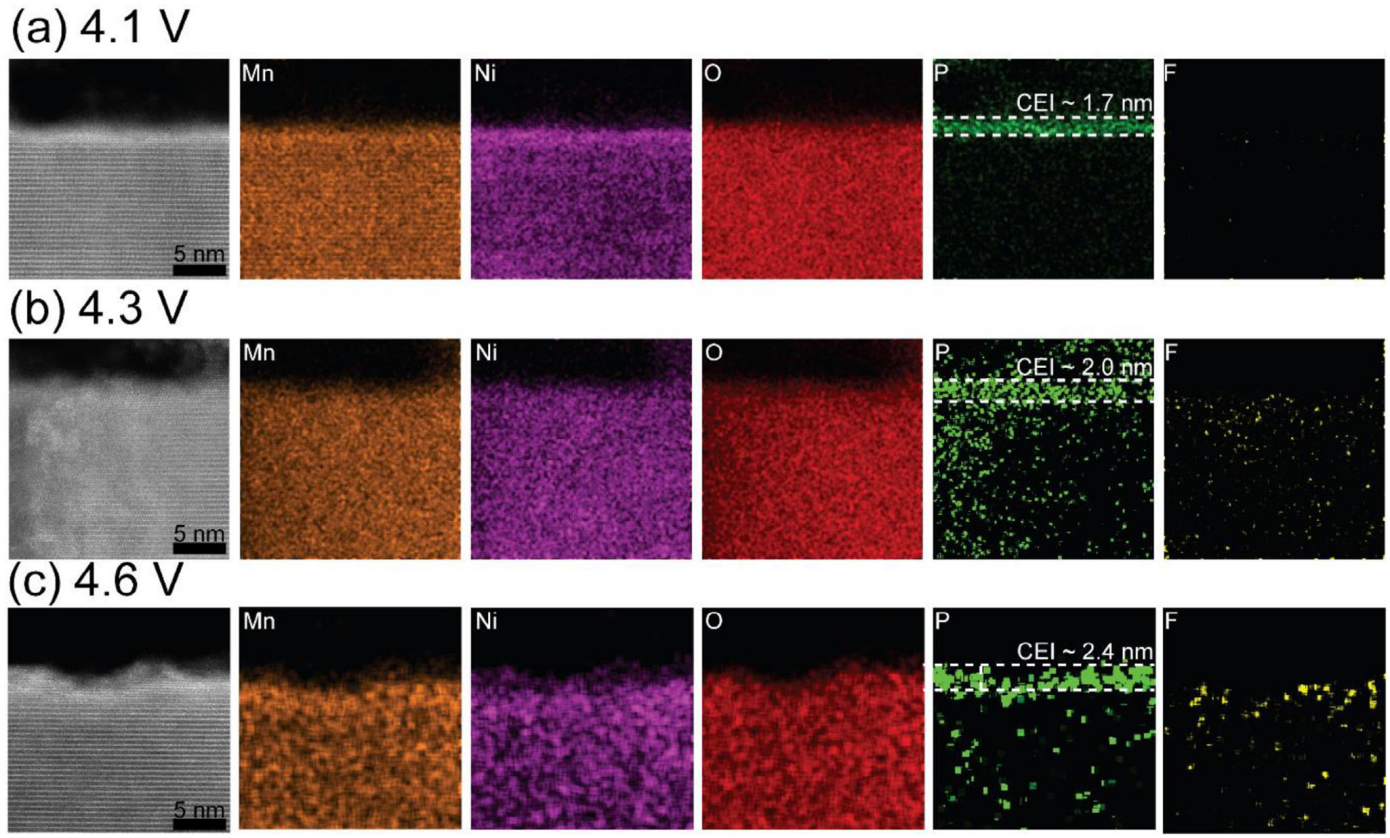
STEM‐EDS elemental maps showing CEI layer formation on LMR37 samples after three initial cycles between (a) 2.5–4.1 V, (b) 2.5–4.3 V and (c) 2.5–4.6 V.

The interactions between the cathode surface and the electrolyte exhibit a clear dependence on the activation voltage. Higher upper cut‐off voltages lead to more pronounced cathode–electrolyte reactions, as reflected by the formation and growth of the cathode–electrolyte interphase (CEI) layer shown in Figure 6. A P‐rich CEI layer is present on the surface of all cathode particles after three cycles between 2.5–4.1, 2.5–4.3, and 2.5–4.6 V. However, both the CEI thickness and the elemental *P*/*F* ratio vary systematically with cycling voltage. As shown in Figure 6, increasing the upper cut‐off voltage results in a thicker CEI layer after the same number of cycles, approximately 1.7 nm at 4.1 V, 2.1 nm at 4.3 V, and 2.4 nm at 4.6 V. This trend indicates that higher activation voltages intensify the surface reactions between the LMR cathode and the electrolyte, consistent with the increased oxygen loss and transition‐metal reduction observed from the STEM–EELS results (Figure 5). Quantitative compositional analysis of the CEI layers using STEM–EDS further reveals a strong voltage dependence in the *P*/*F* ratio: approximately 1.3 at 4.1 V, 0.4 at 4.3 V, and 0.5 at 4.6 V. These results suggest that distinct side reactions dominate at different voltages.

Given that the electrolyte contains LiPF_6_ salts (see Experimental Section), the chemical composition of the CEI layers indicates that electrolyte decomposition occurs even during the initial formation cycles. Moreover, the data suggest that F‐containing, insoluble CEI species are preferentially generated at higher voltages, while P‐containing phases dominate at lower voltages. This voltage‐dependent trend is also evident from the STEM–EDS spectra in Figure S3. Together, these findings show that the choice of activation voltage influences both the thickness and chemical makeup of the CEI layer, which can subsequently affect the interfacial stability and long‐term electrochemical performance of LMR cathodes.

Recently, it has been demonstrated that solid‐electrolyte interphase (SEI) shows voltage dependent electron leakage behavior [38]. A high voltage will lead to continued electron leaking through SEI layer and therefore a thick SEI layer. It would be expected that CEI layer should show similar voltage dependent electron leakage behavior, which could give clues as why a higher voltage activation of cathode will lead to a thicker CEI layer as observed here.

### 2.5 Mechanistic Implications of Activation‐Driven Phase Transformation

Recent studies have shown that in situ formed spinel‐like phases in lithium‐rich DRX cathodes exhibit high capacity and stable cycling [32]. In this context, the present observation of a spinel‐like phase emerging from the *C2/m* domains of LMR oxides during high‐voltage activation is particularly significant. These findings clarify the essential role of electrochemical activation in improving LMR performance and explain why a sufficiently high activation voltage is necessary to initiate structural transformation.

This work identifies that a critical activation voltage exists, above which TM migration and atomic rearrangement are triggered within the *C2/m* lattice for the spinel‐like phase formation, yet such atomic migration mechanism has not been established. Consistent with literature, the Li_2_MnO_3_‐type layered phase is thermodynamically less stable than the spinel structure; thus, TM ions tend to migrate into Li layers during cycling, promoting partial reconstruction of the structure [29]. Furthermore, tetrahedral sites are energetically more favorable than octahedral sites under high‐voltage conditions, explaining why the spinel‐like phase forms only during high‐voltage activation when Li ions gain sufficient energy to occupy tetrahedral positions. Indeed, previous studies have shown that Li ions preferably go into the tetrahedral sites first and then to the octahedral sites with increasing the Li content [39, 40].

Atomic‐scale analyses further reveal that TM migration occurs preferentially from sites in the layered structure that are equivalent to the 16d sites of the *Fd* $\bar{3}$ *m* spinel framework, as indicated by stronger image intensity relative to the 16c sites. However, the overall intensity at 16d sites remains lower than expected for a fully ordered spinel, suggesting incomplete TM substitution within Li layers. The resulting structure is therefore best described as a *spinel‐like* phase rather than a fully developed spinel, in agreement with atomic‐resolution studies reporting partial TM migration and local spinel‐like ordering in Li‐rich oxides [7, 22, 23]. The formation of this spinel‐like phase within Li‐rich *C2/m* domains further supports its close relationship with Li‐rich chemistry, consistent with transformations reported for Li‐rich DRX and lithiated spinel systems [22, 32].

Importantly, the spinel‐like phase observed here arises from a bulk lattice transformation, not from the surface reconstruction processes commonly seen in Ni‐rich layered oxides [34]. In typical surface reconstructions, oxygen loss and lithium depletion yield thin TM_3_O_4_ or TM–O rock‐salt layers, where TM represents transition metals. In contrast, the present spinel‐like domains originate within the bulk *C2/m* lattice without major oxygen deficiency, as supported by correlated STEM‐EELS and structural analyses. Such spinel‐like phase formation is attributed to lattice re‐organization caused by the highly mobile Mn ions in the *C2/m* domains, where a critical voltage is needed [32]. It should be pointed out that due to the limited detection volume of STEM imaging for identifying spinel‐like phase, characterization techniques such as x‐ray diffraction and Raman spectroscopy would be useful in probing the spinel‐like phase on the average level. More detailed work needs to be carried out.

Although voltage fading remains a key challenge for LMR cathodes, the layered‐to spinel‐like transformation does not directly alleviate it, as Li occupancy at tetrahedral sites does not significantly increase. Nonetheless, the enhanced structural robustness of the spinel‐like framework offers a valuable direction for next‐generation cathode design, inspiring efforts to deliberately synthesize or stabilize such phases in bulk form within the research community.

## 3 Conclusions

Electrochemical activation has long been recognized as a critical step for achieving the optimal performance of lithium‐ and manganese‐rich (LMR) cathodes. In this study, we directly reveal that electrochemical activation above a threshold voltage of ∼4.6 V, induces the formation of a spinel‐like phase originating from the *C2/m* domains of the layered LMR structure but not in the *R* $\bar{3}$ *m* domains. Below this voltage, the layered configuration remains largely preserved, indicating that the transformation is voltage‐dependent and requires sufficient driving force for transition‐metal migration.

Atomic‐resolution STEM imaging and the TEM SAED analyses indicate that the newly formed spinel‐like phase maintains crystallographic coherence with the parent layered structure, sharing a continuous oxygen sublattice. The phase is structurally distinct from the ideal cubic spinel (*Fd* $\bar{3}$ *m*) phase, featuring partial occupation of the vacant 16c octahedral site and a mixed occupation of Li and TM at the 8a and 16d sites. Complementary EELS and EDS analyses further show that higher activation voltages promote oxygen loss, transition‐metal reduction, and thicker, more F‐rich cathode–electrolyte interphase (CEI) layers, underscoring the coupled effects of voltage on both bulk and interfacial transformations.

The discovery that a bulk spinel‐like phase forms within the LMR lattice, rather than a surface reconstruction, clarifies the mechanistic basis for electrochemical activation and its role in stabilizing the cathode structure during subsequent cycling. Given recent reports that spinel‐like domains can enable reversible redox activity and improved cycling stability in Li‐rich disordered rock‐salt materials, the coexistence of layered and spinel‐like domains in activated LMR cathodes likely contributes to their enhanced durability.

Overall, these findings provide direct mechanistic insight into the activation process in LMR cathodes, establishing the critical role of high‐voltage induced structural reorganization in determining long‐term electrochemical stability. Understanding and controlling this activation‐driven transformation will be essential for optimizing the next generation of high‐energy, structurally resilient Li‐rich cathodes.

## Conflicts of Interest

The authors declare no conflict of interest.

## Supporting information




**Supporting File 1**: anie71943‐sup‐0001‐SuppMat.docx.

## Data Availability

The data that support the findings of this study are available from the corresponding author upon reasonable request.
