# Supplementary material for: Origin of Electrochemical Activation Leading to Enhanced Cycling Stability of Li‐ and Mn‐Rich Cathodes
Source: Angew Chem Int Ed Engl. 2026 Mar 23;65(19):e8818196. doi: 10.1002/anie.8818196 (PMC13134597; doi:10.1002/anie.8818196)
Supplement: Supplementary file 1 — Supporting File 1: anie71943‐sup‐0001‐SuppMat.docx. [file ANIE-65-e8818196-s001.docx]

*Supporting Information*

**Origin of Electrochemical Activation Leading to Enhanced Cycling Stability of Li- and Mn-Rich Cathodes**

Peng Zuo^[a]^*, Daniel P. Abraham^[b]^*, and Chongmin Wang^[a]^*

[a] Peng Zuo, Chongmin Wang
Environmental Molecular Sciences Laboratory, Pacific Northwest National Laboratory, 902 Battelle Boulevard, Richland, WA 99354, USA
E-mail: ([pzuo@purdue.edu](mailto:pzuo@purdue.edu); [chongmin.wang@pnnl.gov](mailto:chongmin.wang@pnnl.gov))

[b] Daniel P. Abraham
Chemical Sciences and Engineering Division, Argonne National Laboratory, 9700 South Cass Avenue, Lemont, IL 60439, USA
E-mail: ([abraham@anl.gov](mailto:abraham@anl.gov))

1. **Experimental Section**

The 0.3Li_2_MnO_3_·0.7LiMn_0.5_Ni_0.5_O_2_ (LMR37) oxide particles were synthesized at Argonne National Laboratory as described previously.[15] In brief, a Mn_0.65_Ni_0.35_CO_3_ precursor was first synthesized in a continuous stirred-tank reactor (CSTR) and subsequently lithiated with Li_2_CO_3_ to obtain the desired LMR37 composition. The electrodes, fabricated at the CAMP facility (Argonne), comprised a coating of these oxide particles (92 wt%), polyvinylidene difluoride (PVdF) binder (6 wt%) and carbon conduction additives (4 wt%) on a “primed” Al foil current collector.

All LMR37 samples examined in this study are from full cells: in these, the LMR37 electrodes were coupled with anodes comprising a coating of graphite (92 wt%), PVdF (4 wt%) and carbon black (2 wt%) on a Cu foil current collector. These battery cells also contained a Celgard 2500 separator and an electrolyte comprising 1.2 M LiPF_6_ in EC:EMC (3:7 w/w) with 1 wt% LiBF_2_(C_2_O_4_) as the additive. Electrochemical cycling was performed on a MACCOR Series 4000 system with cells housed in a temperature chamber maintained at 30 °C.

Cross-sectional specimens for scanning transmission electron microscopy (STEM) were prepared using a focused ion beam (FIB) technique. A FEI Helios Hydra UX FIB–SEM (Thermo Fisher Scientific) equipped with a Xe plasma source was used to lift out thin lamellae for STEM analysis. The plasma beam energy during sample preparation was gradually reduced from 30 kV to 5 kV to minimize beam damage.

STEM studies were performed on an aberration-corrected Spectra Ultra transmission electron microscope (TEM) (Thermo Fisher Scientific, Inc.) operated at 300 kV. High-angle annular dark-field (HAADF) images were collected over a detector angle range of 68–200 mrad. The STEM annular dark-field (STEM-ADF) images were collected in the 27−162 mrad range. STEM–energy-dispersive X-ray spectroscopy (STEM–EDS) using an Ultra-X detector (Thermo Fisher Scientific) operated at 300 kV. STEM-EELS data were obtained using a Gatan Continuum 1069HR image filter.


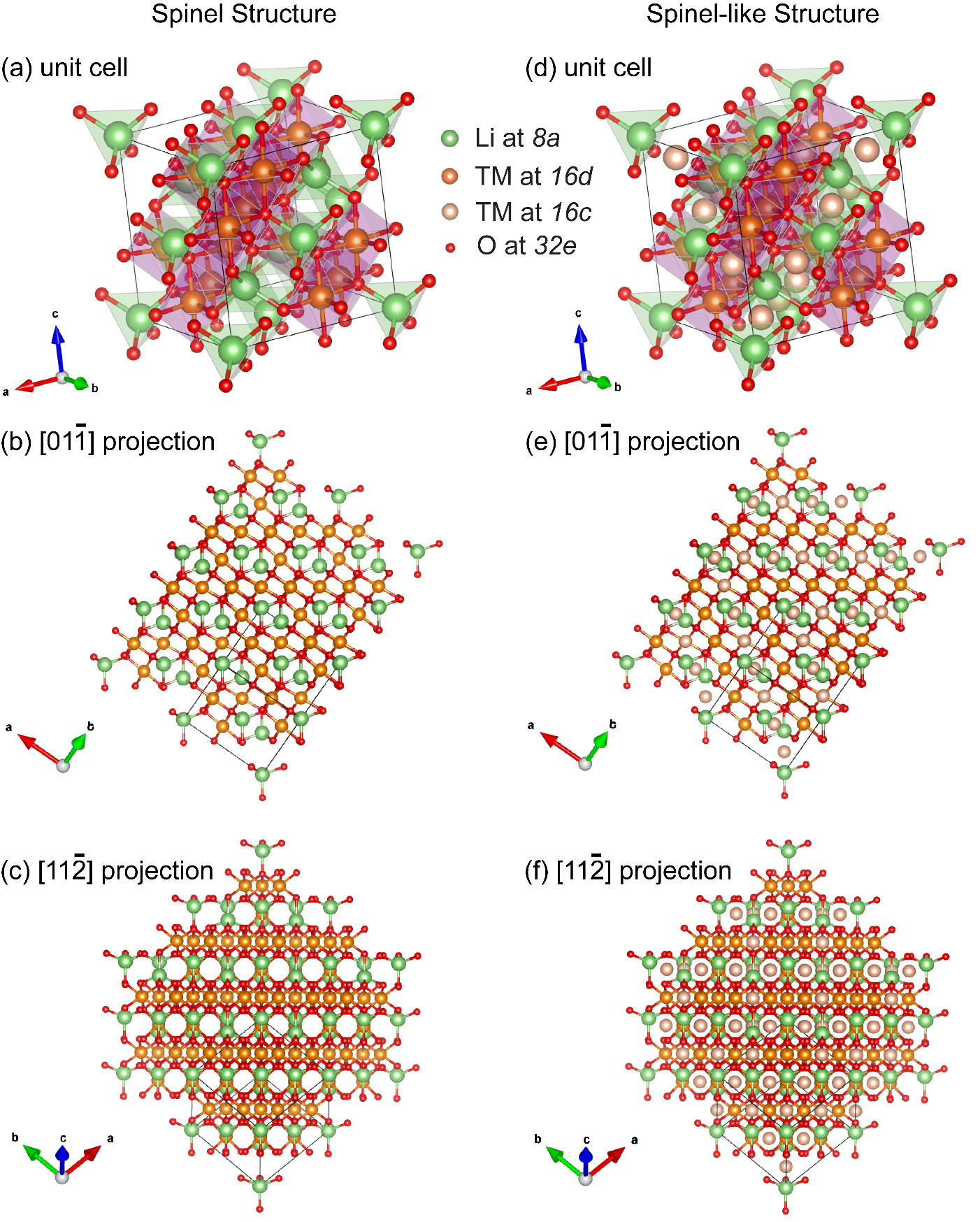


**Figure S1.** A comparison of the structural models of the spinel and spinel-like phase. (a) the spinel structure unit cell, (b) the project along the [01$\bar{1}$] zone axis and (c) the projection along the [11$\bar{2}$] zone axis; (d) the spinel-like structure unit cell, (e) the project along the [01$\bar{1}$] zone axis and (c) the projection along the [11$\bar{2}$] zone axis, where transition metals also present at the *16c* site of the *Fd*$\bar{3}$*m* structure.


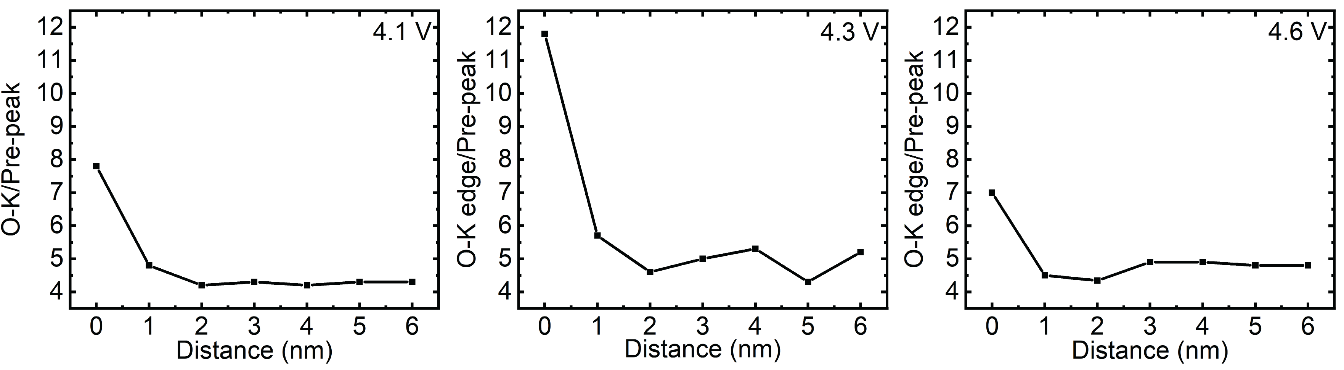


**Figure S2.** The intensity ratio between the O K edge and its pre-peak in STEM-EELS for particles cycled at different voltages 2.5- 4.1 V, 2.5 - 4.3 V and 2.5 - 4.6 V, at the particle edge as a function of distance to the particle interior.


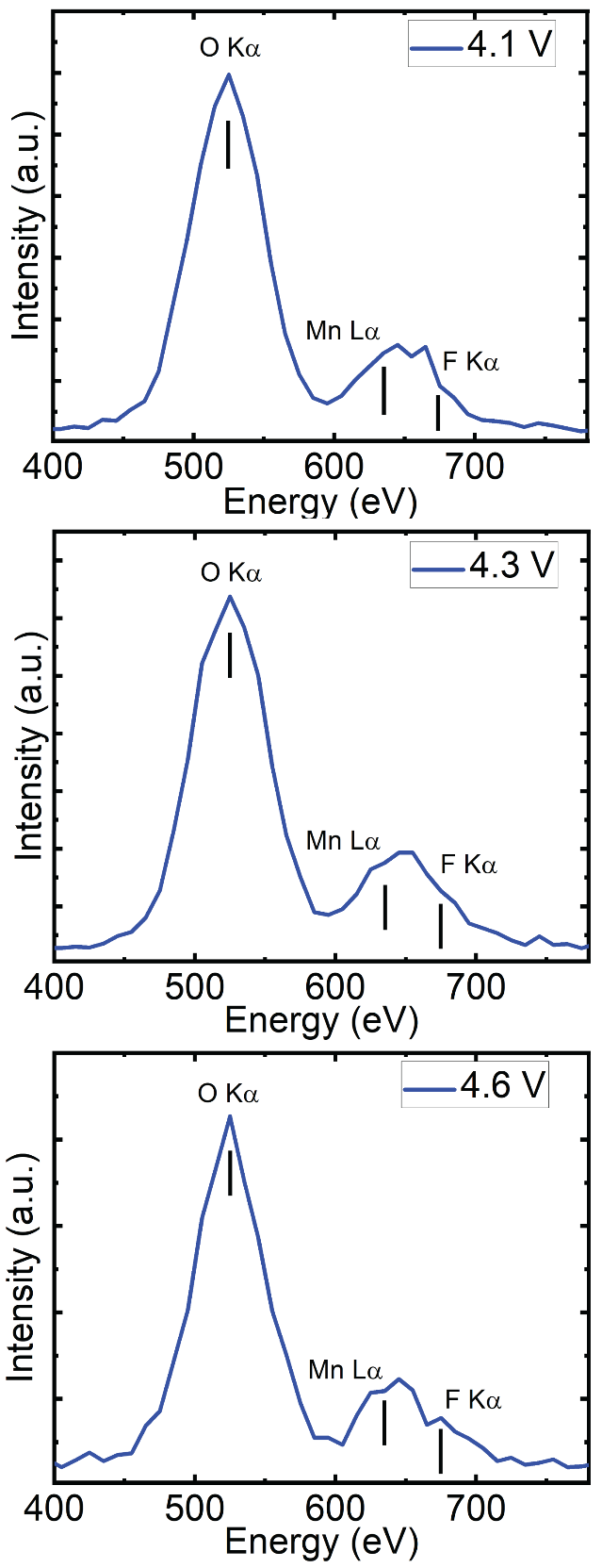


**Figure S3.** The STEM-EDS spectra reconstructed from the CEI layers of the particles cycled at 2.5 - 4.1 V, 2.5 - 4.3 V and 2.5 - 4.6 V, respectively.
